# Orthognathic surgery and postoperative nausea and vomiting: An 11-year retrospective observational study

**DOI:** 10.1371/journal.pone.0346972

**Published:** 2026-04-20

**Authors:** Takuma Watanabe, Ryuji Uozumi, Tatsuya Kawamura, Michinobu Sasaki, Risa Okada, Shu Inoue, Marina Kashiwagi, Shizuko Fukuhara, Shigeki Yamanaka, Sayaka Mishima, Akihiko Yamaguchi, Makoto Hirota

**Affiliations:** 1 Department of Oral and Maxillofacial Surgery, Graduate School of Medicine, Kyoto University, Kyoto, Japan; 2 Department of Industrial Engineering and Economics, Institute of Science Tokyo, Tokyo, Japan; 3 Department of Oral and Maxillofacial Surgery, Kyoto City Hospital, Kyoto, Japan; 4 Department of Oral and Maxillofacial Surgery, Takashima Municipal Hospital, Shiga, Japan; College of Dentistry, University of Anbar, IRAQ

## Abstract

Orthognathic surgery (OGS) is now widely performed, and postoperative nausea and vomiting (PONV) remains a common complication. Gastric decompression using a nasogastric tube (NT) is generally considered an effective prophylactic measure. In this retrospective observational study, we reviewed 667 OGS cases in 632 patients treated at our department between 2014 and 2024 to evaluate the role of NT placement in PONV by examining associations between clinical variables and PONV. Variables were extracted from electronic medical records, and descriptive statistics and annual trends were summarized. Univariable and multivariable logistic regression analyses were performed to examine associations with PONV. The mean age was 27.2 years, and 462 cases (69.3%) involved female patients. Single-jaw surgery was performed in 363 cases (54.4%) and double-jaw surgery in 304 cases (45.6%). Postoperative NT placement was used in 278 cases (41.7%), and PONV occurred in 53 cases (7.9%). The annual number of OGS cases increased from 48 in 2014 to 123 in 2024. Multivariable logistic regression analysis showed that age ≥ 30 years (odds ratio (OR), 0.43; 95% confidence interval (CI), 0.19–0.95; p = 0.04) and ondansetron administration (OR, 0.21; 95% CI, 0.04–0.97; p = 0.05) were associated with a lower risk of PONV, whereas postoperative NT placement was associated with a higher risk (OR, 4.03; 95% CI, 1.74–9.32; p = 0.001). These findings suggest that the number of OGS cases may be increasing, possibly reflecting growing societal awareness. In addition, postoperative NT placement may increase the risk of PONV, particularly in younger patients, while ondansetron may be effective in reducing PONV.

## Introduction

Jaw deformities are multifactorial conditions resulting from congenital, developmental, and acquired factors that disrupt normal craniofacial growth. Genetic variations affecting maxillofacial development contribute to skeletal discrepancies and malocclusion [1]. Trauma is also an important etiological factor, as mandibular fractures may lead to secondary deformities when displacement or malunion occurs [2]. In clinical practice, jaw deformities encompass these diverse etiologies and therefore require comprehensive evaluation and interdisciplinary management, often involving surgical interventions such as orthognathic surgery (OGS) [3].

OGS is widely performed to correct congenital and acquired dentofacial discrepancies, with implications for masticatory function and facial esthetics [4]. Since the inclusion of surgical orthodontic treatment in Japan’s National Health Insurance coverage in 1990 and its subsequent social recognition, OGS has been carried out in many Japanese institutions [5]. Advances in surgical techniques, safer anesthetic agents, and modern monitoring devices have reduced the risk of life-threatening complications in OGS [6]. Nevertheless, postoperative nausea and vomiting (PONV) remains one of the most frequent and distressing complications, contributing to patient dissatisfaction [7–10].

The etiology of PONV after OGS is multifactorial, involving patient-, surgical-, and anesthesia-related factors [10]. Female sex, age under 25 years, high body mass index, nonsmoking status, bimaxillary surgery, and procedures lasting more than 3 hours have been implicated as risk factors [7,8,10–13]. A previous retrospective study reported that 40% of patients experienced PONV within the first 24 hours after OGS [7], while another found high rates of postoperative nausea (67%) and vomiting (27%) [8]. However, no standardized protocol has yet been established that effectively prevents PONV following OGS [11].

Regarding prophylactic measures against PONV, gastric decompression using a nasogastric tube (NT) is generally considered by anesthesiologists to be effective [9,14]. Some researchers suggest that it facilitates the removal of residual blood from the stomach, whereas others argue that an indwelling tube may act as a chronic irritant to the pharynx and stomach, potentially provoking PONV through glossopharyngeal nerve stimulation [8,12–15]. Thus, the role of a NT in PONV remains controversial [13], and it is still unclear whether it reduces the incidence of PONV [16].

In this observational study, we retrospectively reviewed 11 years of OGS cases at our department to investigate associations among patient-, surgical-, anesthetic-, and complication-related factors. Based on our clinical experience, we hypothesized that postoperative NT placement would affect the incidence of PONV. This study aimed to evaluate the role of NT placement in PONV after OGS by analyzing associations between individual variables and PONV using multivariable logistic regression to identify strategies for effective prevention.

## Materials and methods

### Study design and cohort

This study included 667 surgical cases from 632 patients who underwent OGS at the Department of Oral and Maxillofacial Surgery, Kyoto University Hospital. The discrepancy between the number of cases and patients reflects the fact that some patients underwent more than one procedure.

### Inclusion and exclusion criteria

The inclusion criteria were OGS underwent between January 1, 2014, and December 31, 2024. The exclusion criterion was incomplete follow-up data due to a lack of complete computerized information.

### Data collection

Data were retrospectively obtained from electronic medical records. The access to database to obtain the data used in this study was on May 11, 2025. The authors had no access to information that could identify individual participants during or after data collection. Collected variables were categorized into patient-, surgical-, anesthetic- and complication-related factors. Patient-related variables included sex, age, body mass index (BMI) at the initial visit, skeletal diagnosis, the presence of cleft lip and palate (CLP), and smoking status. Surgical-related variables included type of procedure, intraoperative blood loss, operating time, and whether a NT was postoperatively placed. Anesthetic-related variables included the perioperative administration of desflurane, sevoflurane, propofol, fentanyl, remifentanil, ondansetron, and dexamethasone. Complication-related variables included foreign body, angular cheilitis, epistaxis, and nausea and vomiting. Nausea and vomiting was defined as the primary outcome variable. Although postoperative nausea and vomiting are biologically distinct phenomena, in accordance with previous studies, we considered them as a single event in this study and referred to it as PONV [13]. During surgery, all patients received throat packs as a physical barrier against blood and irrigation fluids. Most patients were followed for one year after OGS.

### Statistical analysis

Descriptive statistics for patient-, surgical-, anesthetic- and complication-related variables were presented in tables as numbers and percentages. The annual number of cases, intraoperative blood loss, operating time, presence or absence of postoperative NT placement, and number of cases with PONV were summarized in figures. Univariable and multivariable logistic regression analyses were conducted to examine the associations between each variable and the outcome (PONV), and present odds ratios (ORs) and 95% confidence intervals (CIs) from both analyses are presented in tables. Variables included in the multivariable logistic regression model were selected based on the clinical expertise of oral and maxillofacial surgeons and the results of the univariable logistic regression analysis. All statistical analyses were conducted using JMP Student Edition 18 (SAS Institute, Cary, NC, USA).

### Ethical approval

This study was performed in accordance with the Declaration of Helsinki and was approved by the Institutional Review Board of Kyoto University Hospital (R4257). All patients’ records and information were anonymized and de-identified prior to analysis. The need for patient consent was waived owing to the retrospective study design and data anonymization. Consent for this study was obtained using the opt-out method with descriptions of the study on hospital’s website. Participants had the option to opt out of the study after viewing study information shared via hospital’s website.

## Results

### Descriptive analysis of variables

**Table 1** summarizes the characteristics of the variables. Among the patient-related variables, 205 cases (30.7%) were male and 462 cases (69.3%) were female, with a mean age of 27.2 years (range, 15–59 years) and a mean BMI of 20.9 kg/m² (range, 13.5–40.5 kg/m²). Patients younger than 30 years accounted for 71.8% of all cases, while those with a BMI < 25 kg/m² accounted for 91.8%. The most common skeletal classification was Class 3 (65.2%). There were 9 cases with CLP, and 54 cases (8.1%) were smokers.

**Table 1:** Characteristics of the study cases (n = 667).

Patient-related variables			
Sex			
Male		205	(30.7)
Female		462	(69.3)
Age (years)			
Range			15–59
Mean±SD			27.2 ± 8.3
<30		479	(71.8)
≥30		188	(28.2)
BMI (kg/m^2^)			
Range			13.5-40.5
Mean±SD			20.9 ± 2.9
<25		612	(91.8)
≥25		55	(8.2)
Skeletal Class			
1		82	(12.3)
2		150	(22.5)
3		435	(65.2)
Cleft Lip and Palate			
(-)		658	(98.7)
(+)		9	(1.3)
Smoking			
(-)		613	(91.9)
(+)		54	(8.1)
Surgical-related variables			
Procedure			
Single-Jaw		363	(54.4)
	Bilateral SSRO	190	(28.5)
	Bilateral IVRO	87	(13.0)
	Genioplasty	32	(4.8)
	AMSO	23	(3.4)
	SSRO/IVRO	14	(2.1)
	Others	17	(2.6)
Double-Jaw		304	(45.6)
	LF1 + Bilateral SSRO	206	(30.9)
	LF1 + SSRO/IVRO	30	(4.5)
	LF1 + Bilateral IVRO	28	(4.2)
	AMSO + Bilateral SSRO	18	(2.7)
	Others	22	(3.3)
Blood Loss (ml)			
	Range	0-2590	
	Median (Interquartile Range)	220 (95-420)	
Operating Time (min)			
	Range	46-528	
	Median (Interquartile Range)	213 (152-297)	
Nasogastric Tube			
	(-)	389	(58.3)
	(+)	278	(41.7)
Anesthetic-related variables			
Desflurane			
	(-)	422	(63.3)
	(+)	245	(36.7)
Sevoflurane			
	(-)	397	(59.5)
	(+)	270	(40.5)
Propofol			
	(-)	48	(7.2)
	(+)	619	(92.8)
Fentanyl			
	(-)	319	(47.8)
	(+)	348	(52.2)
Remifentanil			
	(-)	7	(1.0)
	(+)	660	(99.0)
Ondansetron			
	(-)	444	(66.6)
	(+)	223	(33.4)
Dexamethasone			
	(-)	550	(82.5)
	(+)	117	(17.5)
Complication-related variables			
Foreign Body			
	(-)	663	(99.4)
	(+)	4	(0.6)
Angular Cheilitis			
	(-)	662	(99.3)
	(+)	5	(0.7)
Epistaxis			
	(-)	653	(97.9)
	(+)	14	(2.1)
Nausea and Vomiting			
	(-)	614	(92.1)
	(+)	53	(7.9)

Data are presented as n (%) unless otherwise indicated.

SD = standard deviation; BMI = body mass index; LF1 = Le Fort Ⅰ osteotomy; SSRO = sagittal split ramus osteotomy; IVRO = intraoral vertical ramus osteotomy; AMSO = anterior maxillary segmental osteotomy

Regarding surgical-related variables, 363 procedures (54.4%) were single-jaw surgeries and 304 (45.6%) were double-jaw surgeries. Le Fort Ⅰ osteotomy + bilateral sagittal split ramus osteotomy (SSRO) was the most common procedure (206 cases, 30.9%), followed by bilateral SSRO (190 cases, 28.5%). The median intraoperative blood loss was 220 mL (interquartile range, 95–420 mL), and the median operating time was 213 minutes (interquartile range, 152–297 minutes). A NT was postoperatively placed in 278 cases (41.7%).

Regarding anesthetic-related variables, desflurane, sevoflurane, or propofol was administered perioperatively in 245 cases (36.7%), 270 cases (40.5%), and 619 cases (92.8%), respectively. Fentanyl and remifentanil were used as opioids in 348 cases (52.2%) and 660 cases (99.0%), respectively. Ondansetron and dexamethasone was administered as an antiemetic in 223 cases (33.4%) and 117 cases (17.5%), respectively.

With respect to complication-related variables, intraoperatively, foreign body migration occurred in 4 cases (0.6%). Postoperatively, 5 cases (0.7%) developed angular cheilitis, 14 cases (2.1%) experienced epistaxis, and 53 cases (7.9%) experienced nausea and vomiting.

### Annual number of cases

**Fig 1:**
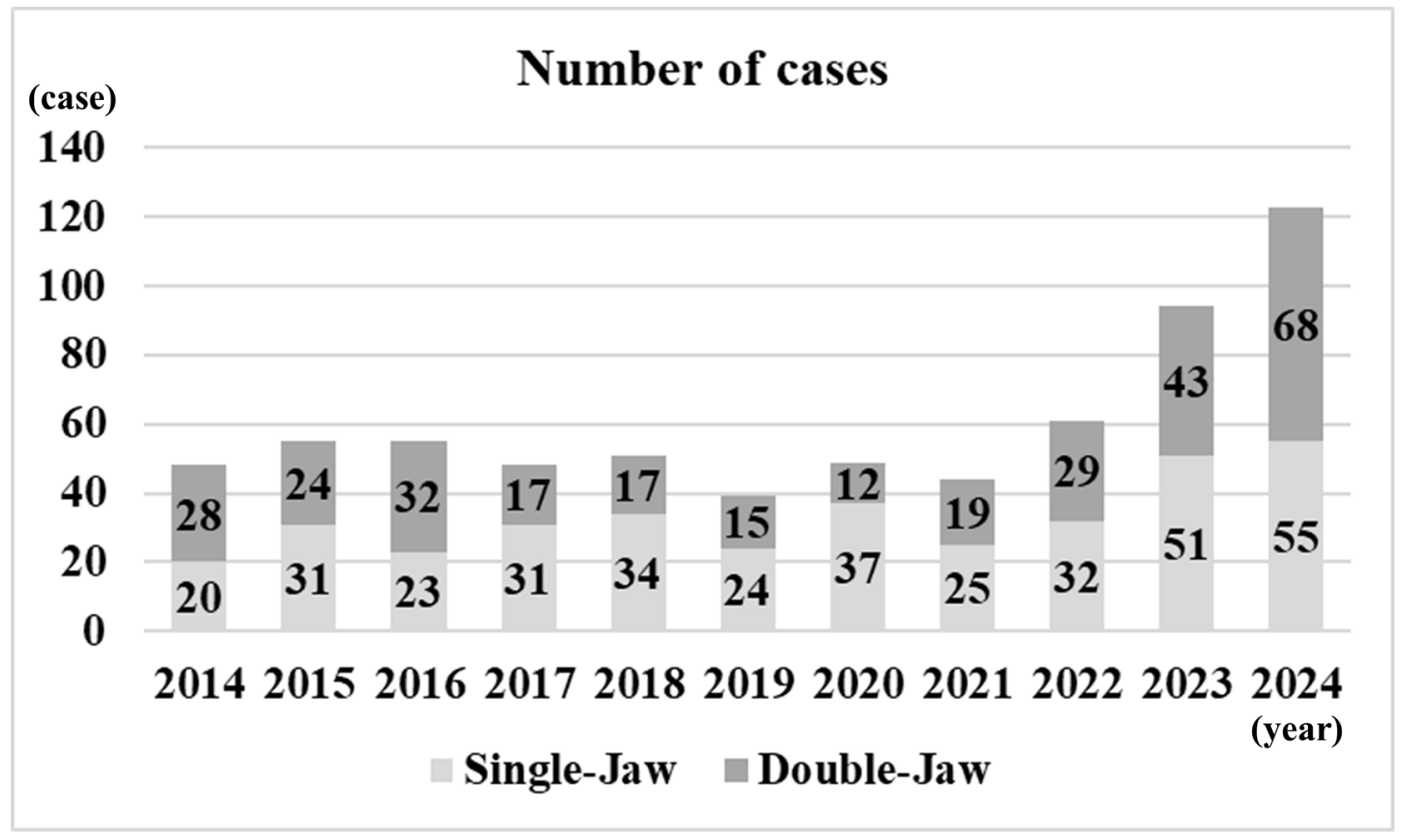
Number of cases per year.

**Fig 2:**
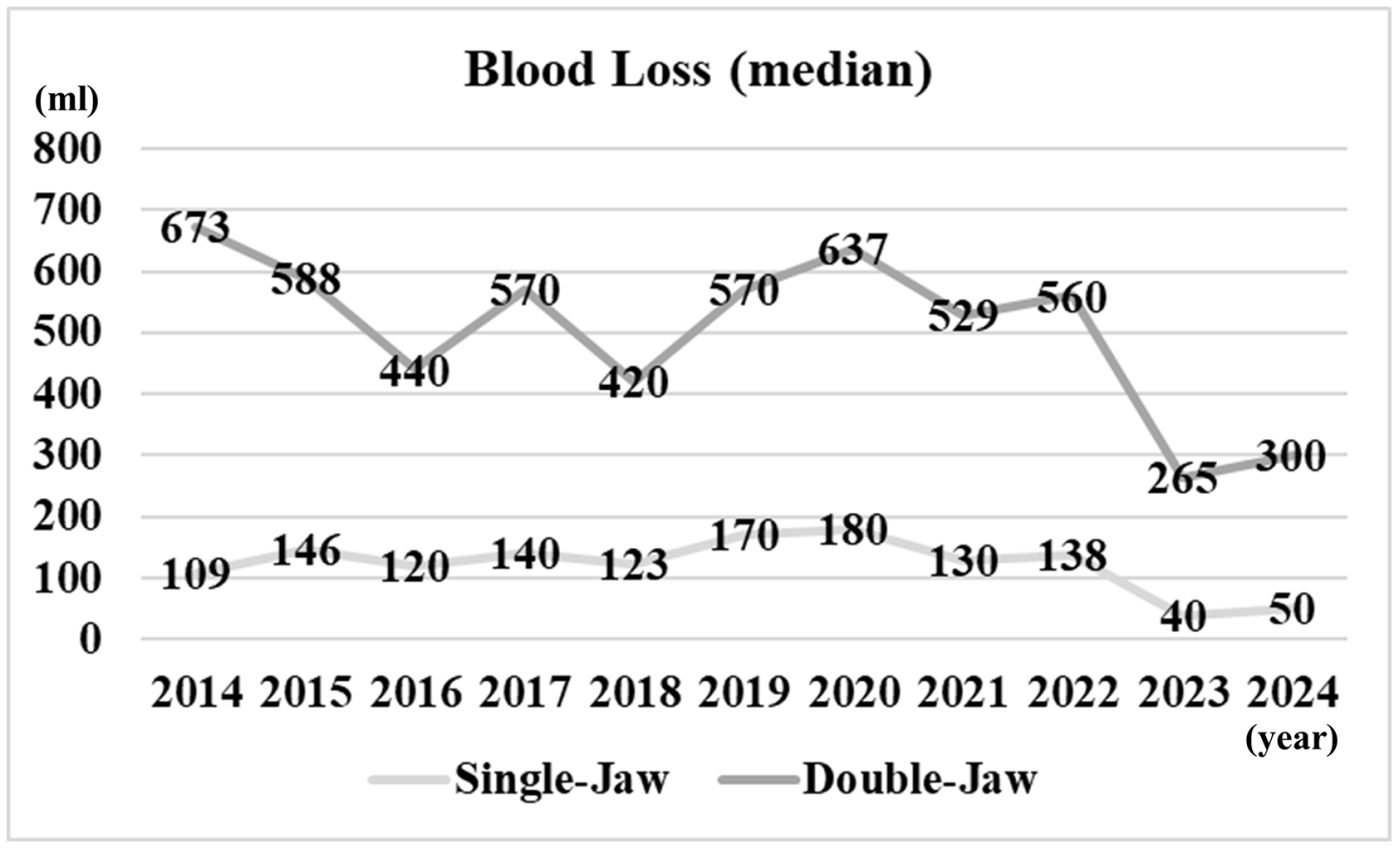
Median intraoperative blood loss per year.

**Fig 1** shows the annual number of OGS cases, including single-jaw and double-jaw procedures. In 2014, there were 20 single-jaw cases and 28 double-jaw cases, whereas in 2024, the numbers increased to 55 and 68, respectively. **Fig 2** shows the median intraoperative blood loss per year, stratified by single-jaw and double-jaw procedures. For single-jaw procedures, the median blood loss decreased from 109 mL in 2014 to 50 mL in 2024. For double-jaw procedures, it decreased from 673 mL in 2014 to 300 mL in 2024.

**Fig 3:**
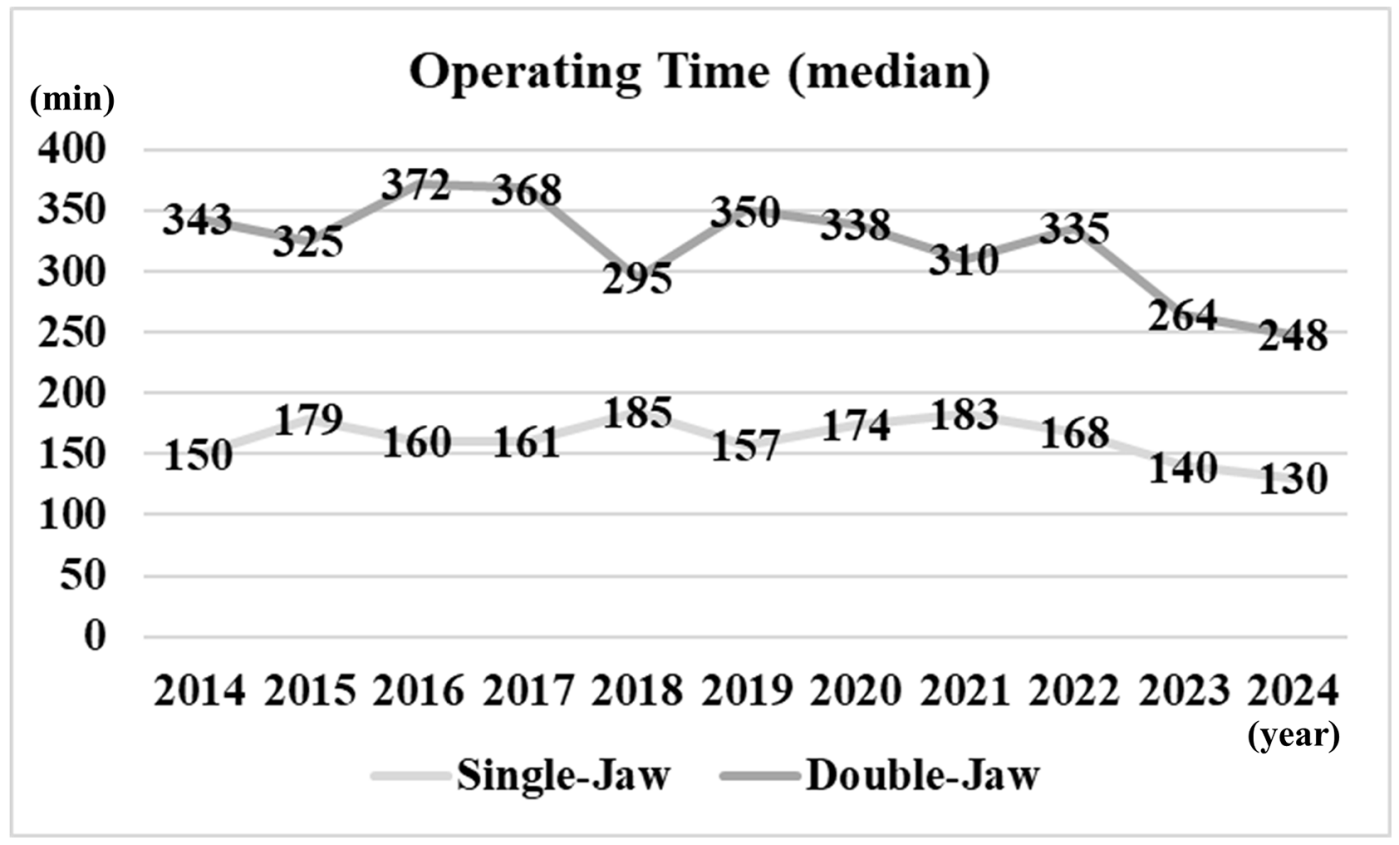
Median operating time per year.

**Fig 4:**
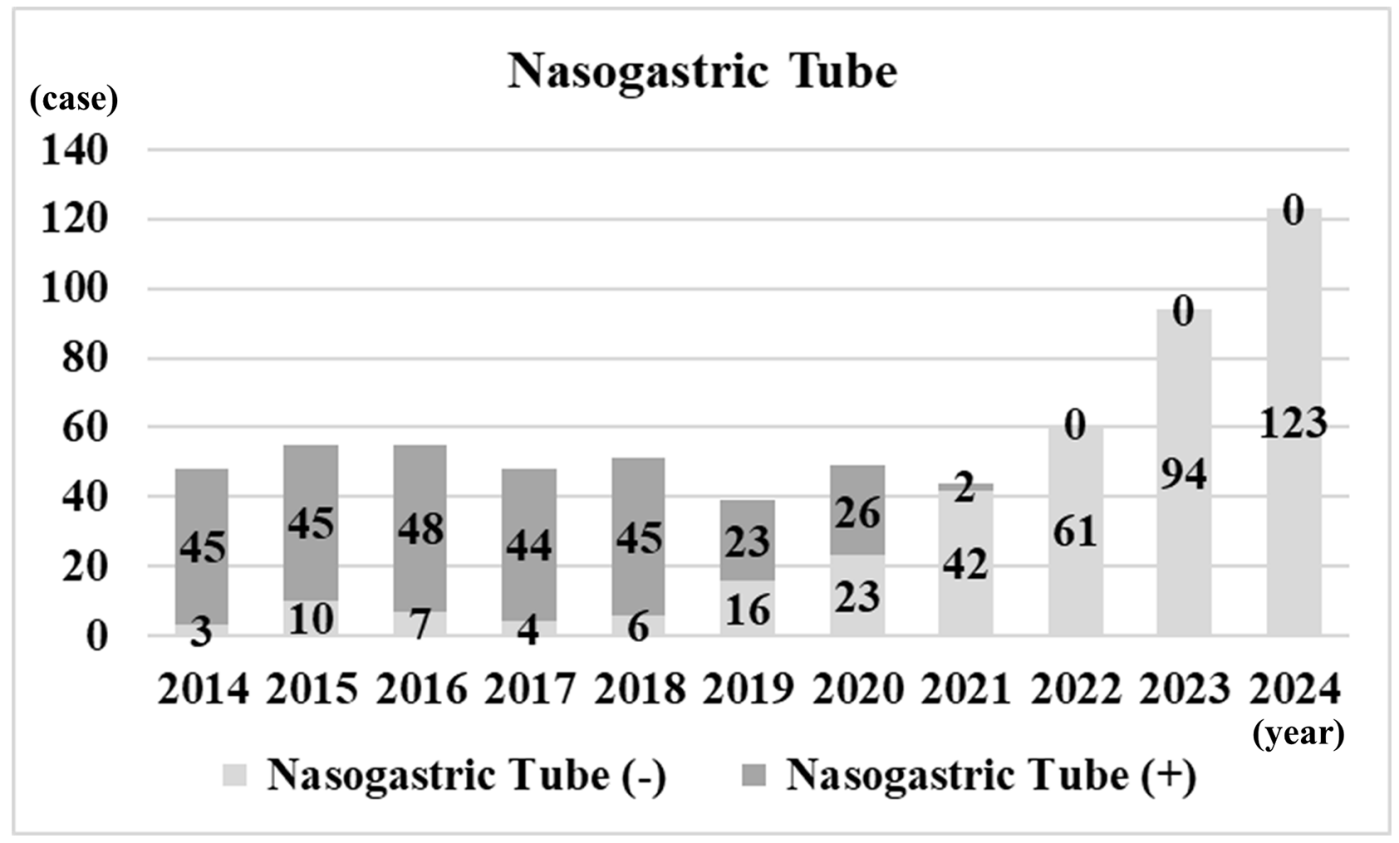
Number of cases with postoperative NT placement per year.

**Fig 5:**
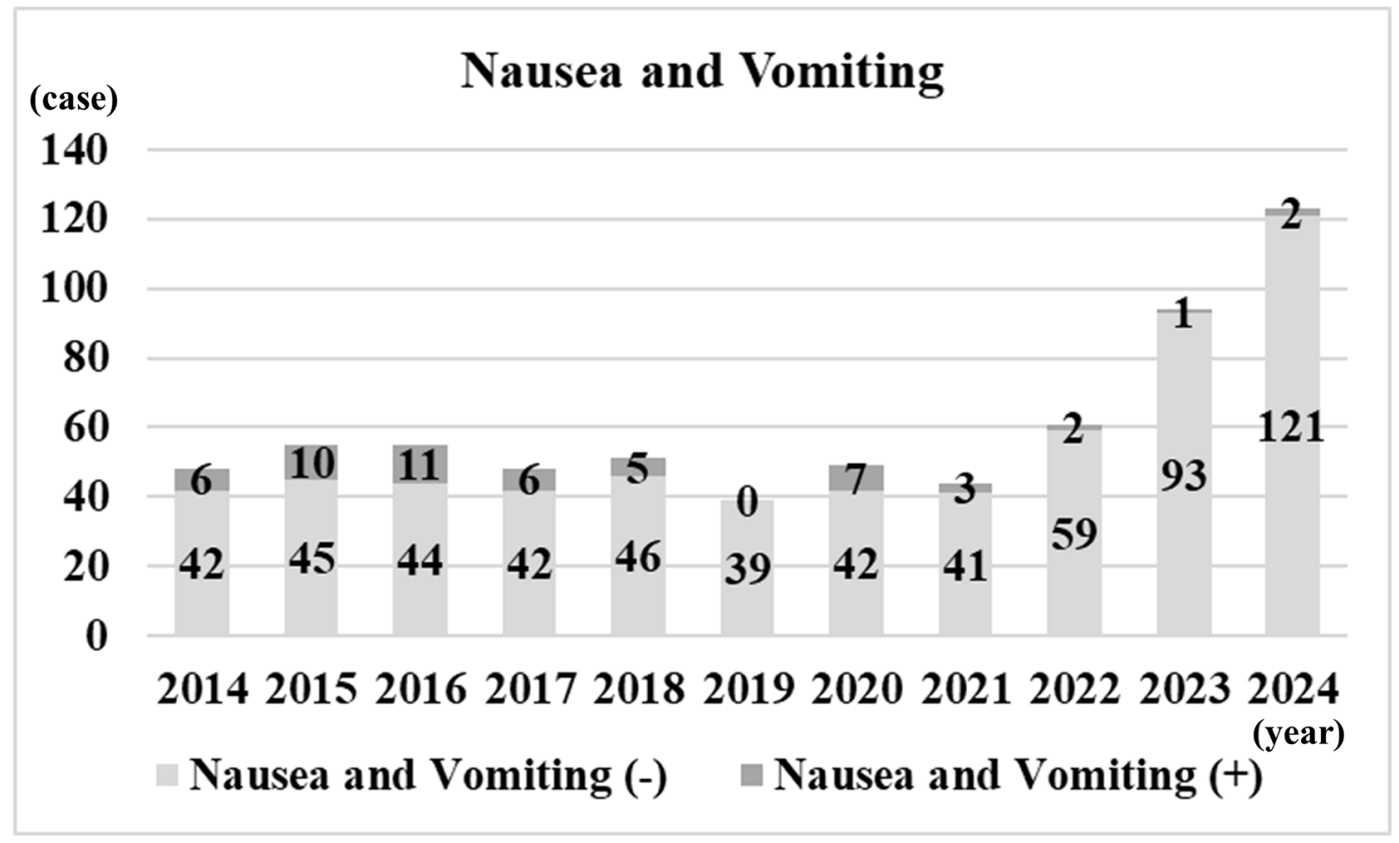
Number of cases with PONV per year.

**Fig 3** shows the median operating time per year, stratified by single-jaw and double-jaw procedures. For single-jaw procedures, the median operating time decreased from 150 minutes in 2014 to 130 minutes in 2024. For double-jaw procedures, it decreased from 343 minutes in 2014 to 248 minutes in 2024. **Fig 4** shows the annual number of cases with and without postoperative NT placement. In 2016, NTs were postoperatively placed in 48 cases, but no placements have been performed since 2022. **Fig 5** shows the annual number of cases with PONV. PONV was most prevalent in 2016 with 11 cases, whereas in 2023 it was least prevalent with only 1 case.

### Logistic regression analyses for PONV

**Table 2** summarizes the univariable and multivariable logistic regression analyses conducted to examine the associations between each variable and PONV. Univariable logistic regression analysis showed that PONV was associated with age (ref: < 30 years; OR, 0.43; 95% CI, 0.18–0.88; p = 0.02), procedure (ref: Single-Jaw; OR, 1.76; 95% CI, 1.00–3.14; p = 0.05), blood loss (ref: < 220 ml; OR, 1.82; 95% CI, 1.03–3.33; p = 0.04), operating time (ref: < 213 min; OR, 2.01; 95% CI, 1.13–3.70; p = 0.02), postoperative NT placement (ref: no; OR, 6.12; 95% CI, 3.20–12.70; p < 0.001), desflurane (ref: no; OR, 2.63; 95% CI, 1.50–4.71; p < 0.001), ondansetron (ref: no; OR, 0.11; 95% CI, 0.03–0.30; p < 0.001), and dexamethasone (ref: no; OR, 0.36; 95% CI, 0.11–0.91; p = 0.03). Multivariable logistic regression analysis indicated that age ≥ 30 years was associated with a lower risk of PONV (OR, 0.43; 95% CI, 0.19–0.95; p = 0.04), whereas postoperative NT placement was associated with an increased risk of PONV (OR, 4.03; 95% CI, 1.74–9.32; p = 0.001). In addition, ondansetron was also associated with a decreased risk of PONV (OR, 0.21; 95% CI, 0.04–0.97; p = 0.05).

**Table 2:** Univariable and multivariable logistic regression analyses to determine the independent predictor of nausea and vomiting.

Variables	Univariable			Multivariable		
	OR (95%CI)		p-value	OR (95%CI)		p-value
Sex (ref: Male)	1.14	(0.62, 2.18)	0.69			
Age (ref: < 30)	0.43	(0.18, 0.88)	0.02	0.43	(0.19, 0.95)	0.04
BMI (ref: < 25)	0.65	(0.15, 1.85)	0.45			
Skeletal Class 2 (ref: Class 1)	0.74	(0.32, 1.80)	0.50			
Skeletal Class 3 (ref: Class 1)	0.51	(0.25, 1.15)	0.10			
Skeletal Class 3 (ref: Class 2)	0.69	(0.36, 1.39)	0.29			
Cleft Lip and Palate (ref: [-])	3.40	(0.50, 14.51)	0.18			
Smoking (ref: [-])	0.92	(0.27, 2.38)	0.88			
Procedure (ref: Single-Jaw)	1.76	(1.00, 3.14)	0.05	1.71	(0.63, 4.62)	0.29
Blood Loss (ref: < 220)	1.82	(1.03, 3.33)	0.04	0.73	(0.31, 1.72)	0.48
Operating Time (ref: < 213)	2.01	(1.13, 3.70)	0.02	1.53	(0.56, 4.20)	0.41
Nasogastric Tube (ref: [-])	6.12	(3.20, 12.70)	<0.001	4.03	(1.74, 9.32)	0.001
Desflurane (ref: [-])	2.63	(1.50, 4.71)	<0.001	1.72	(0.94, 3.18)	0.08
Sevoflurane (ref: [-])	0.74	(0.40, 1.32)	0.31			
Propofol (ref: [-])	2.07	(0.61, 12.87)	0.28			
Fentanyl (ref: [-])	1.57	(0.89, 2.83)	0.12			
Remifentanil (ref: [-])	0.51	(0.09, 9.78)	0.57			
Ondansetron (ref: [-])	0.11	(0.03, 0.30)	<0.001	0.21	(0.04, 0.97)	0.05
Dexamethasone (ref: [-])	0.36	(0.11, 0.91)	0.03	2.61	(0.65, 10.44)	0.17

ref = reference; OR=odds ratio; CI = confidence interval; BMI = body mass index.

## Discussion

In this study, we retrospectively reviewed 11 years of OGS cases at our department to examine associations among patient-, surgical-, anesthetic-, and complication-related factors, with a particular focus on PONV. Our findings demonstrated a substantial increase in the annual number of OGS procedures over the study period, rising from 48 cases in 2014 to 123 cases in 2024. Notably, 53 cases (7.9%) experienced PONV, and multivariable logistic regression analysis revealed that age ≥ 30 years and administration of ondansetron was associated with a reduced risk of PONV, whereas postoperative NT placement was associated with an increased risk.

With the increased social recognition of the surgical orthodontic treatment of jaw deformities in Japan, the number of patients undergoing OGSs has increased [5]. The increase in OGS may be attributed to greater demand, improved marketing, advances in techniques, procedures, and tools, and more predictable outcomes [5,17]. It has now become an important field of oral and maxillofacial surgery [5]. Mandibular protrusion is a common characteristic in the Japanese population [5], whereas the higher prevalence among female patients may reflect their desire for cosmetic improvement, since OGS addresses many aspects of facial esthetics [5]. Single-jaw surgery is less invasive and more predictable than double-jaw surgery, but is potentially inadequate at achieving a harmonious profile and occlusion in cases of mandibular protrusion, which is prevalent among Japanese patients [5]. In our study, similar to previous report, most patients were young females with a Class 3 skeletal pattern. The annual number of OGS procedures markedly increased, with approximately half of the cases involving single-jaw surgery.

Intraoperative bleeding is one of the most serious complications of OGS, and it may arise from the maxillary artery and vein, the pterygoid arteriovenous plexus, retromandibular vein, the palatine artery, or the facial artery [4]. Surgery duration can be prolonged by the need for additional procedures or by the involvement of residents in each case [8]. In our department, as part of surgical training, procedures are performed by multiple practitioners with varying levels of experience. Nevertheless, a decreasing trend in both operating time and blood loss has been observed over time. This trend may be attributed to favorable educational outcomes and the use of safer instruments, such as piezosurgery devices. In recent years, we have also performed OGS under hypotensive general anesthesia in cooperation with anesthesiologists, which may have further contributed to this decline.

Independent factors associated with PONV can be categorized into patient-related (gender, age, smoking status), surgical-related (type of surgery, blood loss, duration, NT), and anesthetic-related (volatile anesthetics, intraoperative opioid use, antiemetic medication) factors [6–13,16]. Although the incidence of PONV is influenced by these factors and has been reported to vary among centers [6,7,13,16], the incidence observed in our study was relatively low at 7.9%.

Regarding the association between age and PONV, previous studies have shown that younger patients are more likely to experience PONV, with its incidence decreasing as age increases [7]. One study reported that the likelihood of PONV decreases by 13% for every 10-year increase in age [18], while another found that patients younger than 30 years had a higher incidence of PONV compared with older patients [16]. Consistent with these findings, our study demonstrated that younger age was strongly associated with the occurrence of PONV.

As for the association between surgical procedures, blood loss, operating time, and PONV, these factors may influence the occurrence of PONV [7,9,11,13]. Previous studies have shown a greater number of emetic episodes when the maxilla is involved, with the highest frequency of PONV observed in the double-jaw surgery group [7]. It has been hypothesized that increased swallowing of blood and, potentially, deliberate hypotension contribute to this higher incidence of PONV [7]. Maxillary procedures are typically associated with greater intraoperative bleeding, swallowing of blood, and the use of hypotensive anesthesia, all of which increase the risk of PONV [13]. Prolonged surgeries also lead to greater exposure to anesthetic agents that can induce PONV and are therefore associated with a higher risk [9]. Unlike these previous studies, the results of multivariable analysis in this study showed that procedures, blood loss, and operating time were not strongly associated with PONV.

Gastric aspiration can be a simple and safe procedure that reduces the amount of fluid in the stomach, including blood, during the immediate postoperative period following OGS [6]. A previous randomized clinical trial demonstrated that gastric aspiration after OGS was effective in reducing PONV [6]. In contrast, a previous prospective study investigating gastric aspiration in patients undergoing OGS found no difference in the overall incidence of PONV, although the sample size was small [10]. Moreover, a NT may even increase the incidence of PONV by stimulating the glossopharyngeal nerve [7]. In addition, consensus guidelines have stated that the use of a NT has been disproven as a method for reducing PONV [8,12]. At our department, prior to 2019, NT insertion and gastric aspiration via the tube before extubation were routinely performed in OGS patients to reduce the amount of blood in the stomach. In addition, the NT was typically left in place for a few days postoperatively for enteral feeding. However, because many patients complained of pharyngeal discomfort and nausea associated with the use of a NT, its postoperative use has been discontinued since 2022. The observed association between postoperative NT placement and PONV should be interpreted with caution, as residual and temporal confounding may be present. Nevertheless, our findings are consistent with our clinical observations, suggesting that postoperative NT placement may be associated with an increased occurrence of PONV, whereas intraoperative gastric aspiration via the NT may help reduce gastric contents.

General anesthesia can be administered using intravenous (IV) agents and/or inhaled volatile anesthetics. A previous prospective, randomized, multicenter trial demonstrated that, compared with inhalational anesthesia, total IV anesthesia reduced the incidence of PONV during the early postoperative period [19]. Among inhaled volatile anesthetics, comparative study have shown a much lower incidence of PONV with isoflurane (4%) compared with desflurane (67%) and sevoflurane (36%), which may be attributed to the higher solubility of isoflurane relative to the other agents [20]. In the present study, the use of desflurane was not observed to be associated with an increased incidence of PONV in the multivariable analysis.

The administration of antiemetic drugs for the prevention or treatment of PONV is an important measure that should be appropriately utilized [7]. Both dexamethasone and ondansetron have been shown to be effective in preventing PONV; therefore, the use of aggressive antiemetic prophylaxis at the end of surgery is recommended [11]. A scoping review concluded that prophylactic antiemetics such as dexamethasone and ondansetron represent the first line of defense against PONV [10]. A recent prospective randomized controlled trial reported that the combination of dexamethasone and ondansetron was more effective than dexamethasone alone in preventing early postoperative nausea and reducing the need for rescue antiemetics for PONV in patients undergoing OGS [21]. At our department, although not in all case, dexamethasone has been administered to OGS patients since 2017, while ondansetron has been used since 2022. Although the administration of these drugs has only recently been introduced into our clinical practice, our study demonstrated that ondansetron was associated with a reduced occurrence of PONV in the multivariable analysis.

The main limitation of this study is its retrospective design. The analysis relied on the accuracy and completeness of data available in the electronic medical records, which may have introduced the risk of missing data due to gaps in information or incomplete documentation. Secondly, this study was conducted at a single institution, which may limit the generalizability of the findings. Third, data regarding maxillomandibular fixation (MMF), which can exacerbate the anxiety and agitation associated with PONV, were not obtained. Previously, MMF with wires was performed for a few days postoperatively. However, in recent years, we have discontinued postoperative MMF with wires because stable occlusion can now be achieved owing to improvements in surgical techniques and accuracy.

## Conclusion

This retrospective single-center study provides an overview of the characteristics of patients who underwent OGS at our department over an eleven-year period. The primary objective was to describe trends in OGS cases and to examine patient-, surgical-, and anesthetic-related factors associated with PONV. Our findings show an increase in the number of OGS cases at our department during the study period, which may reflect growing societal recognition of surgical orthodontic treatment. In addition, postoperative NT placement may be associated with an increased incidence of PONV, suggesting that avoidance of postoperative NT placement in younger patients may help reduce the risk of PONV. The administration of ondansetron may be effective in controlling PONV following OGS. Further multicenter, prospective studies with larger cohorts are needed to clarify these associations and to establish strategies for improving patient satisfaction, including effective PONV prevention, as the number of OGS procedures continues to increase across institutions.
